# Prevention of new-onset chronic kidney disease with renin–angiotensin system blockers in type 2 diabetes with preserved kidney function

**DOI:** 10.1080/0886022X.2026.2711459

**Published:** 2026-08-05

**Authors:** Wan-Chia Hsu, Shih-Yuan Hung, Sheng-Hsiang Lin, Yuan-Yow Chiou, Yu-Ling Lin, Hsi-Hao Wang, Li-Chun Ho, Yi-Che Lee

**Affiliations:** a Division of Endocrinology and Metabolism, Department of Internal Medicine, Kaohsiung Chang Gung Memorial Hospital, Kaohsiung, Taiwan; b School of Medicine, College of Medicine, I-Shou University, Kaohsiung, Taiwan; c Division of Nephrology, Department of Internal Medicine, E-DA Hospital, Kaohsiung, Taiwan; d Institute of Clinical Medicine, College of Medicine, National Cheng Kung University, Tainan, Taiwan; e Department of Public Health, College of Medicine, National Cheng Kung University, Tainan, Taiwan; f Biostatistics Consulting Center, National Cheng Kung University Hospital, College of Medicine, National Cheng Kung University, Tainan, Taiwan; g Department of Pediatrics, National Cheng Kung University Hospital, Tainan, Taiwan; h Division of Nephrology, Department of Internal Medicine, Hsinchu Cathay General Hospital, Hsinchu, Taiwan

**Keywords:** Angiotensin-converting enzyme inhibitors, angiotensin-receptor blocker, chronic kidney disease, diabetic kidney disease, diabetes mellitus, proteinuria

## Abstract

Renin–angiotensin system (RAS) blockers, including angiotensin-converting enzyme inhibitors (ACEIs) and angiotensin receptor blockers (ARBs), are known to slow chronic kidney disease (CKD) progression in patients with established renal impairment. However, whether RAS blockade can prevent new-onset CKD in patients with type 2 diabetes and hypertension who maintain preserved kidney function remains unclear. We addressed this question in a multicenter retrospective cohort study. Beginning with 316,693 individuals with type 2 diabetes and newly diagnosed hypertension, we retained 3,316 ACEI/ARB users and 1,409 nonusers after generalized boosted model weighting to equalize baseline characteristics. Three renal endpoints were followed: a sustained eGFR below 60 mL/min/1.73 m^2^, incident albuminuria (urinary albumin-to-creatinine ratio of 30 mg/g or higher), and a composite of either. Associations between RAS blocker exposure and each endpoint were quantified using Cox proportional hazards regression, with competing-risk and time-dependent variants applied as confirmatory analyses. ACEI/ARB therapy was associated with a significantly lower risk of new-onset CKD (HR 0.72; 95% CI 0.60–0.86), with consistent findings after accounting for competing risks (HR 0.73; 95% CI 0.61–0.87) and time-dependent exposure (HR 0.81; 95% CI 0.68–0.96). These findings suggest that RAS blockade may confer early kidney protection in patients with type 2 diabetes and hypertension with preserved renal function.

## LAY SUMMARY

People with type 2 diabetes often develop high blood pressure, and together these can slowly damage the kidneys. Medicines known as renin–angiotensin system blockers (angiotensin-converting enzyme inhibitors and angiotensin receptor blockers) are already used once kidney damage is present, but it has been unclear whether they help earlier. Starting from 316,693 people with type 2 diabetes across seven hospitals in Taiwan, we identified those who had normal kidney function and no protein in the urine when blood-pressure treatment was first started, and compared 3,316 who began one of these medicines with 1,409 who began other blood-pressure medicines. Those taking an angiotensin-converting enzyme inhibitor or an angiotensin receptor blocker were about 28% less likely to develop new kidney disease, with the clearest benefit in preventing protein from appearing in the urine, an early sign of kidney injury. These medicines may therefore be linked to kidney protection before damage begins, although a randomized trial is still needed to confirm this.

**KEY MESSAGES**
**What is known:** Renin–angiotensin system blockers are established therapy for slowing kidney function decline in patients with type 2 diabetes who already have a reduced eGFR or albuminuria, but whether they offer any advantage over other antihypertensive classes before kidney injury has occurred remains uncertain, with prior trials yielding inconsistent results.
**This study adds:** Starting from a multicenter database of 316,693 patients with type 2 diabetes, we compared 3,316 ACEI/ARB users with 1,409 nonusers who had preserved kidney function and normoalbuminuria at the initiation of antihypertensive therapy, and found that ACEI/ARB use was associated with a significantly lower risk of new-onset CKD (HR 0.72; 95% CI 0.60–0.86), with the association most robust for incident albuminuria and the composite endpoint.
**Potential impact:** When antihypertensive therapy is first initiated in patients with type 2 diabetes and preserved kidney function, an ACEI or ARB may be a reasonable preferential choice for the primary prevention of albuminuria and new-onset CKD, a hypothesis that now warrants prospective confirmation.

## Introduction

In patients with both diabetes and chronic kidney disease (CKD), characterized by a urinary albumin-to-creatinine ratio (uACR) ≥30 mg/g or an estimated glomerular filtration rate (eGFR) <60 mL/min/1.73 m^2^, angiotensin-converting enzyme inhibitors (ACEIs) or angiotensin II receptor blockers (ARBs) have unequivocally been proven to slow the progression of kidney function decline, and clinical guidelines uniformly recommend prioritizing these agents in this population [1–4].

In contrast, among patients with preserved renal function, defined by an eGFR ≥60 mL/min/1.73 m^2^ and absence of proteinuria, current evidence does not demonstrate that ACEIs or ARBs confer superior renal protection compared with other first-line antihypertensive classes, such as thiazide-like diuretics or dihydropyridine calcium channel blockers [1,5].

Prior studies, including randomized trials, observational research, and meta-analyses, have yielded inconsistent results regarding the efficacy of ACEIs or ARBs in preventing new-onset kidney disease in this subgroup [5–13]. To address this gap, the present study investigates whether the use of ACEIs or ARBs in patients with type 2 diabetes and hypertension but with preserved renal function provides greater protection against the development of new-onset kidney disease compared with other antihypertensive agents.

## Research design and methods

### Study design

We conducted a multicenter, retrospectively designed observational cohort study. Adults with type 2 diabetes mellitus who were started on antihypertensive medication at the participating institutions during the study window were divided into two groups according to whether or not their regimen contained an ACEI or ARB, and the occurrence of new-onset CKD was then compared between these groups over follow-up.

### Database and study sample

Study participants were drawn from the Chang Gung Memorial Hospital system, which comprises seven affiliated hospitals distributed from northern to southern Taiwan, over the period from January 2006 through December 2016. For each patient, de-identified information was retrieved from the electronic health record, encompassing date of birth and sex, ICD-9 and ICD-10-CM diagnostic codes, dispensed medications, and laboratory results. This study was conducted using the same database that our group has previously used to examine the renal effects of metformin, although it addresses a distinct research question, exposure, study population, and set of outcomes [14,15].

All individuals carrying a diagnosis of type 2 DM between January 2006 and December 2016 formed the initial pool (*n* = 316,693) (Supplementary Figure 1). We first removed 135,496 individuals who were never prescribed any medication for hypertension or diabetes during the identification window, on the assumption that they had declined pharmacologic management, did not require it, or were treated elsewhere. From the remainder, we retained only those whose hypertension was newly diagnosed and whose first antihypertensive prescription fell between January 2007 and December 2016 (*n* = 103,085), excluding anyone already receiving antihypertensive treatment before January 2007. The index date was defined as the day of the first antihypertensive prescription within this study window. Patients were then separated into two groups according to whether their initial antihypertensive regimen contained an ACEI or ARB (ACEI/ARB group) or not (non-ACEI/ARB group). Because the DM diagnosis was required to precede the index date, individuals whose type 2 DM was first recorded afterward were dropped. We further excluded those younger than 18 years and those without a baseline eGFR or UACR measurement, and subsequently patients whose baseline eGFR was below 60 mL/min/1.73 m^2^, whose UACR was ≥ 30 mg/g, those with renal transplantation, polycystic kidney disease, or obstructive uropathy, and anyone lacking glycated hemoglobin (HbA1C) values during the identification period. This left 3,316 ACEI/ARB users and 1,409 nonusers. As the final step, a generalized boosted model (GBM) incorporating age, sex, baseline eGFR category, UACR, diabetes duration, comorbidities, and baseline medications was applied to balance covariates between the two groups.

### Covariate adjustment

To improve between-group comparability, covariate balancing was carried out using a GBM approach [14]. The model included age, sex, baseline eGFR category, baseline UACR, duration of diabetes, a range of comorbidities (hypertension, hyperlipidemia, coronary artery disease, cerebrovascular disease, congestive heart failure, gout, nephrolithiasis, chronic hepatitis and cirrhosis, autoimmune disease, chronic lung disease, and malignancy), and the medications in use at baseline (metformin, sulfonylurea, meglitinide, alpha-glucosidase inhibitors [AGIs], thiazolidinediones [TZD], dipeptidyl peptidase-4 [DPP4] inhibitors, insulin, statin, anti-gout agents, calcium-channel blockers [CCB], beta-blockers, diuretics, aspirin, and non-steroidal anti-inflammatory drugs [NSAID]). GLP-1 receptor agonists and SGLT2 inhibitors were not entered, because very few patients were taking them at the index date. Balancing was judged by the absolute standardized mean difference (aSMD) before and after weighting, with values below 0.1 regarded as acceptable, and a Love plot was used to display the aSMD of every baseline variable.

### Data collection

Serum creatinine values were converted to eGFR using the CKD-EPI (CKD Epidemiology Collaboration) equation [15].

### Outcome

Three primary endpoints were assessed: an eGFR that remained below 60 mL/min/1.73 m^2^ for at least three consecutive months; a UACR that stayed at or above 30 mg/g for at least three consecutive months; and a composite endpoint, reached when either the eGFR or the UACR criterion was satisfied over three consecutive months, irrespective of which occurred first.

### Duration of follow-up/censor date

Follow-up time and the point of censoring differed across participants. Individuals who remained in continuous care through the close of the 2016 database were censored at the database end date, whereas those who reached an endpoint earlier were followed only until that event. Patients who died were observed up to their date of death, and those lost to follow-up up to their last recorded clinic visit. In addition, any nonuser who initiated ACEI/ARB therapy during follow-up was censored at the date of that first prescription.

### Statistical analysis

Baseline differences between groups were tested with the χ^2^ test for categorical variables and the unpaired Student’s t-test for continuous variables. Crude hazard ratios (HRs) with 95% confidence intervals (CIs) were obtained from Cox proportional hazards regression, and multivariable Cox models were then constructed to examine whether ACEI/ARB use was associated with prevention of new-onset CKD. Three nested models were specified. Model 1 was adjusted for age, sex, baseline eGFR, baseline UACR, DM duration, comorbidities and ever-use medications that remained imbalanced (aSMD ≥ 0.1), and the laboratory variables systolic and diastolic blood pressure, total cholesterol, low-density and high-density lipoprotein cholesterol, triglyceride, hemoglobin, and glycated hemoglobin. Model 2 was a competing-risk model based on the sub-distribution hazard function, applying the same covariate set and treating death as a competing event. Model 3 was a time-dependent Cox model in which ACEI/ARB exposure was handled as a time-varying covariate. This analytic approach combined GBM weighting with subsequent regression adjustment in a doubly robust manner. Baseline laboratory covariates that were not included in the first-stage GBM balancing procedure, including systolic and diastolic blood pressure, were directly and fully adjusted for in the multivariable outcome models. In addition, comorbidity and medications that remained imbalanced after weighting, defined as an aSMD ≥0.1, including calcium-channel blockers and beta-blockers, were explicitly entered as covariates according to our prespecified adjustment strategy. All analyses were performed in SAS 9.4 (SAS Institute Inc., Cary, NC, USA) and R 3.0.3 (R Foundation for Statistical Computing).

### Sensitivity analysis of blood glucose control

Because renal prognosis is closely related to glycemic management, we performed a sensitivity analysis incorporating HbA1c as a time-dependent covariate in the Cox models. This approach allowed us to account for dynamic changes in HbA1c and other covariates over time when evaluating the association between ACEI/ARB use and renal outcomes.

### Active-comparator analysis: ACEI/ARB versus calcium-channel blockers

Because the non-ACEI/ARB group comprised a heterogeneous mixture of antihypertensive regimens, a direct comparison against this composite reference group may limit the clinical interpretability of the estimated effect. To address this and to reduce potential confounding by indication, we performed an active-comparator analysis restricted to a single, well-defined alternative class. Calcium-channel blockers were selected as the comparator because they were the most frequently prescribed alternative antihypertensive agent in the non-ACEI/ARB group. ACEI/ARB users were compared directly with calcium-channel blocker users (Supplementary Table 2).

### Sensitivity analysis stratified by follow-up duration

Because CKD development may require a prolonged observation period and follow-up duration differed between the two groups, we performed a sensitivity analysis stratified by follow-up duration. Patients were divided at a cut-point of 1.5 years (≤1.5 vs. >1.5 years), and adjusted hazard ratios were estimated separately within each stratum using the same covariate-adjusted model (Supplementary Table 3).

### Sensitivity analysis using baseline covariates only

Because Model 1 adjusted for medications used throughout follow-up—variables measured after baseline that may introduce time-related bias—we performed a sensitivity analysis adjusting for baseline covariates only, to ensure methodological consistency with the cohort design (Supplementary Table 4).

### Sensitivity analysis of potential residual confounder

To gauge how an unmeasured confounder might affect our results, we used the R package ‘obsSens’ [16]. A hypothetical confounder carrying a risk effect comparable to our observed estimate was introduced, and we then varied its prevalence across the ACEI/ARB and non-ACEI/ARB groups to see how the findings shifted.

## Results

### Baseline characteristics

The baseline profiles of the two ACEI/ARB-defined groups are summarized in Table 1. The cohort comprised 3,316 ACEI/ARB users and 1,409 nonusers. Once covariate weighting had been applied, median eGFR stood at 99.6 mL/min/1.73 m^2^ (interquartile range [IQR] 84.0–117.1) among users versus 97.0 mL/min/1.73 m^2^ (IQR 83.9–115.7) among nonusers, while median age was 58.1 years (IQR 50.9–64.9) and 59.3 years (IQR 52.7–65.8), respectively.

**Table 1.** Demographic characteristics of ACEI/ARB and non- ACEI/ARB cohorts.

	Unweighted, n (%)/ median (IQR)			GBM^a^, n (%)/ median (IQR)		
Variables	ACEI/ARB (*N* = 3316)	Non-ACEI/ARB (*N* = 1409)	aSMD	ACEI/ARB	Non-ACEI/ARB	aSMD
Age			0.196			0.128
<45	496 (14.96)	161 (11.43)		595 (13.95)	402 (12.20)	
45-<55	818 (24.67)	299 (21.22)		993 (23.29)	671 (20.37)	
55-<65	1220 (36.79)	538 (38.18)		1617 (37.92)	1262 (38.31)	
≥65	782 (23.58)	411 (29.17)		1059 (24.84)	960 (29.12)	
Median (IQR)	57.70 [50.34,64.51]	59.63 [52.55,65.98]	0.146	58.18 [50.93,64.97]	59.35 [52.73,65.82]	0.070
Male	1881 (56.72)	845 (59.97)	0.066	2482 (58.20)	2035 (61.75)	0.072
Baseline eGFR, mL/min/1.73 m²			0.028			0.038
60-<70	224 (6.76)	92 (6.53)		291 (6.83)	236 (7.16)	
70-<80	387 (11.67)	170 (12.07)		494 (11.58)	368 (11.16)	
80-<90	525 (15.83)	233 (16.54)		686 (16.09)	553 (16.78)	
≥90	2180 (65.74)	914 (64.87)		2794 (65.50)	2138 (64.90)	
Median (IQR)	99.85 [84.18,118.11]	98.20 [83.79,117.36]	0.026	99.69 [84.03,117.18]	97.07 [83.91,115.75]	0.031
Baseline UACR, mg/g	10.60 [6.70,16.90]	10.10 [6.30,16.20]	0.076	10.50 [6.70,16.70]	10.10 [6.60,16.00]	0.050
DM duration (months)	1.38 [0.00,36.16]	11.01 [0.00,53.52]	0.227	2.30 [0.00,39.13]	4.37 [0.00,43.70]	0.050
Follow up time (years)	2.59 [1.38,4.02]	1.48 [0.48,2.88]	0.539	2.59 [1.40,3.99]	1.51 [0.46,2.97]	0.404
Comorbidity						
Hypertension	1034 (31.18)	343 (24.34)	0.153	1292 (30.30)	853 (25.89)	0.098
Hyperlipidemia	1373 (41.41)	603 (42.80)	0.028	1760 (41.26)	1358 (41.22)	0.001
Coronary artery disease	185 (5.58)	111 (7.88)	0.092	257 (6.03)	219 (6.66)	0.026
Cerebrovascular disease	213 (6.42)	79 (5.61)	0.034	273 (6.40)	187 (5.67)	0.031
Congestive heart failure	48 (1.45)	20 (1.42)	0.002	65 (1.52)	50 (1.53)	0.001
Gout	109 (3.29)	39 (2.77)	0.030	131 (3.08)	82 (2.49)	0.036
Nephrolithiasis	46 (1.39)	29 (2.06)	0.052	59 (1.38)	55 (1.65)	0.023
Chronic hepatitis and liver cirrhosis	517 (15.59)	296 (21.01)	0.140	698 (16.36)	573 (17.38)	0.027
Autoimmune disorders	26 (0.78)	17 (1.21)	0.043	29 (0.69)	23 (0.70)	0.002
Chronic lung disease	85 (2.56)	67 (4.76)	0.117	115 (2.70)	111 (3.38)	0.039
Malignant	323 (9.74)	182 (12.92)	0.100	423 (9.93)	322 (9.76)	0.006
Laboratory data						
SBP, ≥ 140 mmHg	1891 (57.67)	599 (42.94)	0.298	2424 (57.45)	1333 (40.79)	0.338
DBP, ≥ 90 mmHg	823 (25.10)	217 (15.56)	0.239	1049 (24.86)	491 (15.03)	0.248
TC, ≥ 200 mg/dL	852 (25.98)	319 (22.90)	0.072	1055 (25.02)	827 (25.58)	0.013
LDL, ≥ 100 mg/dL	1794 (54.65)	689 (49.50)	0.103	2276 (53.88)	1672 (51.77)	0.042
HDL, ≥ 40 mg/dL	2326 (71.35)	1006 (72.95)	0.036	2978 (71.04)	2375 (74.37)	0.075
TG, ≥ 150 mg/dL	1094 (33.42)	406 (29.21)	0.091	1381 (32.80)	895 (27.72)	0.111
Hb, ≥ 12 g/dL	2311 (85.25)	993 (82.34)	0.079	3020 (86.04)	2245 (79.63)	0.171
HbA1C			0.086			0.064
≥ 9%	775 (23.37)	276 (19.59)		966 (22.66)	685 (20.79)	
8%-<9%	539 (16.25)	210 (14.90)		669 (15.69)	486 (14.76)	
7%-<8%	919 (27.71)	420 (29.81)		1188 (27.85)	974 (29.56)	
<7%	1083 (32.66)	503 (35.70)		1442 (33.81)	1149 (34.89)	
Initial medications in the index data						
Metformin, yes	2975 (89.72)	1204 (85.45)	0.130	3808 (89.30)	2897 (87.93)	0.043
Sulfonylurea, yes	1220 (36.79)	507 (35.98)	0.017	1550 (36.35)	1126 (34.18)	0.045
Meglitinide, yes	118 (3.56)	52 (3.69)	0.007	151 (3.54)	107 (3.26)	0.016
AGIs, yes	308 (9.29)	162 (11.50)	0.072	398 (9.33)	322 (9.76)	0.015
TZD, yes	269 (8.11)	135 (9.58)	0.052	334 (7.84)	278 (8.43)	0.022
DPP4 inhibitor, yes	1211 (36.52)	508 (36.05)	0.010	1540 (36.11)	1192 (36.18)	0.001
GLP1, yes	8 (0.24)	2 (0.14)	0.023	9 (0.22)	3 (0.08)	0.037
SGLT2 inhibitor, yes	5 (0.15)	3 (0.21)	0.015	6 (0.14)	5 (0.15)	0.004
Insulin, yes	359 (10.83)	180 (12.78)	0.060	463 (10.85)	386 (11.73)	0.028
ACEI, yes	322 (9.71)	0 (0.00)	–	431 (10.10)	0 (0.00)	–
ARB, yes	3022 (91.13)	0 (0.00)	–	3874 (90.83)	0 (0.00)	–
Statin, yes	1368 (41.25)	592 (42.02)	0.015	1780 (41.73)	1325 (40.21)	0.031
Anti-gout, yes	67 (2.02)	25 (1.77)	0.018	83 (1.94)	64 (1.94)	<0.001
CC blockers, yes	285 (8.59)	498 (35.34)	0.683	615 (14.43)	713 (21.63)	0.188
Beta blockers, yes	336 (10.13)	449 (31.87)	0.554	615 (14.42)	690 (20.96)	0.172
Diuretics, yes	278 (8.38)	185 (13.13)	0.154	384 (9.01)	353 (10.72)	0.057
Aspirin, yes	674 (20.33)	329 (23.35)	0.073	895 (20.98)	698 (21.17)	0.005
NSAID, yes	245 (7.39)	124 (8.80)	0.052	318 (7.46)	273 (8.28)	0.031

Weighted counts represent propensity-score–weighted pseudo-population counts and may not sum to the original sample size.

ACEI denotes angiotensin-converting enzyme inhibitors, ARB angiotensin-receptor blocker, eGFR estimated glomerular filtration rate, SBP systolic blood pressure, DBP diastolic blood pressure, TC total cholesterol, LDL low-density lipoprotein, HDL high-density lipoprotein, TG Triglyceride, Hb hemoglobin, HbA1C glycated hemoglobin, AGIs alpha-glucosidase inhibitors, TZD Thiazolidinediones, DPP4 dipeptidyl peptidase-4, GLP1 glucagon-like peptide-1, SGLT2 sodium-glucose cotransporter type 2, CC blockers calcium channel blockers, and NSAID non-steroidal anti-inflammatory drug.

aSMD: absolute standardized mean difference.

^a^GBM (generalized boosted model) included eGFR (baseline category), UACR, age, sex, DM duration, comorbidities, and initial medication. It is employed by the Twang methodology, relying on a composition of tree-based regression models built iteratively.

^b^SBP data had 51 missing values, DBP data had 51 missing values, TC data had 52 missing values, LDL data had 50 missing values, HDL data had 86 missing values, TG data had 62 missing values, and Hb data had 808 missing values.

Before weighting, a number of covariates were appreciably unbalanced (aSMD >0.1), as displayed in Supplementary Figure 2. Age, sex, baseline eGFR and UACR, diabetes duration, comorbidities, and baseline medications were all entered into the GBM to harmonize the two groups. Weighting brought the GBM-included variables into much closer alignment, pulling most prespecified covariates under the 0.1 aSMD threshold and many beneath 0.05.

### Incidence of eGFR persistently below 60 mL/min/1.73 m^2^

**Figure 1.**
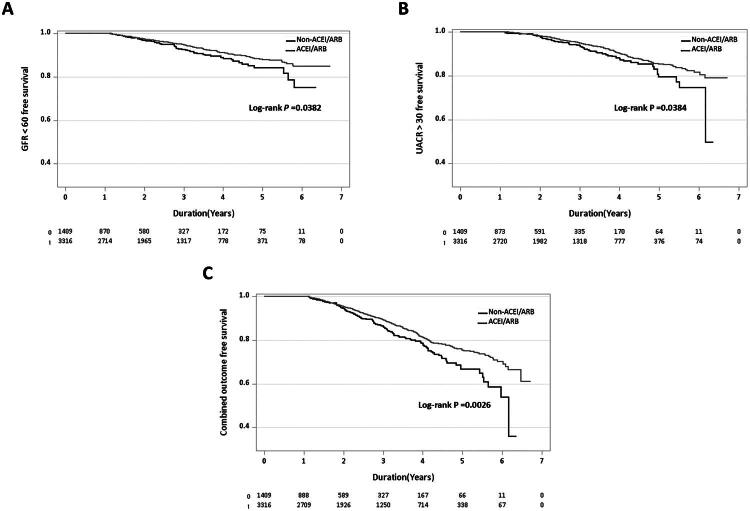
Kaplan–Meier curves for (A) eGFR < 60 mL/min/1.73 m^2^ (B) UACR ≥ 30 mg/g and (C) composite outcome. Between-group differences were assessed by the log-rank test.

Findings for a persistently reduced eGFR (<60 mL/min/1.73 m^2^) are detailed in Table 2. This endpoint arose in 237 patients overall—175 among ACEI/ARB users and 62 among nonusers—across the observation window. Multivariable Cox regression showed the risk to be significantly lower in users (Model 1: HR 0.72; 95% CI 0.56–0.94), and the competing-risk model yielded an essentially identical estimate (Model 2: HR 0.74; 95% CI 0.56–0.97). When exposure was instead treated as time-varying, however, the effect weakened and lost significance (Model 3: HR 1.05; 95% CI 0.82–1.35). The Kaplan–Meier curves likewise separated significantly for cumulative sustained eGFR decline (*p* = 0.038; Figure 1A).

**Table 2.** Adjusted hazard ratios (HRs) of renal outcome among the cohort of sampled patients during the follow-up years.

Type of treatment	No. of events	Incidence rate per 100 patient-years	Study outcome, HR (95% CI)
(No. of Patients)			
eGFR < 60 mL/min/1.73 m^2^			
Nonuser (*n* = 1409)	62	2.34	
ACEI/ARB user (*n* = 3316)	175	1.97	0.86 (0.69–1.06)
Model 1^a^			0.72 (0.56–0.94)
Model 2^b^			0.74 (0.56–0.97)
Model 3^c^			1.05 (0.82–1.35)
UACR ≥ 30 mg/g			
Nonuser (*n* = 1409)	64	2.42	
ACEI/ARB user (*n* = 3316)	192	2.16	0.87 (0.71–1.08)
Model 1^a^			0.65 (0.50–0.85)
Model 2^b^			0.65 (0.49–0.87)
Model 3^c^			0.77 (0.60–0.99)
Composite outcome			
Nonuser (*n* = 1409)	130	4.91	
ACEI/ARB user (*n* = 3316)	359	4.15	0.81 (0.70–0.94)
Model 1^a^			0.72 (0.60–0.86)
Model 2^b^			0.73 (0.61–0.87)
Model 3^c^			0.81 (0.68–0.96)

^a^Adjusted for age, sex, baseline eGFR, baseline UACR, DM duration, comorbidity (including variables with aSMD ≥ 0.1), medication ever used throughout the entire follow-up period (including variables with aSMD ≥ 0.1) and laboratory data.

^b^The Sub-Distribution Hazard Function was adjusted for age, sex, baseline eGFR, baseline UACR, DM duration, comorbidity, laboratory data, and medication, with death considered a competing risk.

^c^Time-dependent Cox models treating the utilization of ACEIs/ARBs as a time-dependent variable.

### Incidence of sustained UACR levels of 30 mg/g or higher

Results for sustained albuminuria (UACR ≥30 mg/g) are likewise shown in Table 2. A total of 256 individuals reached this endpoint during follow-up, distributed as 192 users and 64 nonusers. After multivariable adjustment, ACEI/ARB use again carried a markedly lower risk than nonuse (Model 1: HR 0.65; 95% CI 0.50–0.85), and this protective association held in both the competing-risk (Model 2) and time-dependent (Model 3) analyses. Consistent with these models, the cumulative incidence of albuminuria onset diverged significantly between groups on Kaplan–Meier analysis (*p* = 0.038; Figure 1B).

### Incidence of new onset CKD

The composite new-onset CKD endpoint—met when either the eGFR or the albuminuria criterion was first satisfied—is presented in Table 2. It occurred in 489 patients in total, comprising 359 users and 130 nonusers over the study period. Multivariable Cox regression linked ACEI/ARB use to a significantly reduced risk relative to nonuse (Model 1: HR 0.72; 95% CI 0.60–0.86), with the competing-risk (Model 2) and time-dependent (Model 3) models returning broadly concordant results. The between-group gap in cumulative composite incidence was also significant by Kaplan–Meier analysis (*p* = 0.002; Figure 1C).

### Subgroup analysis

**Figure 2.**
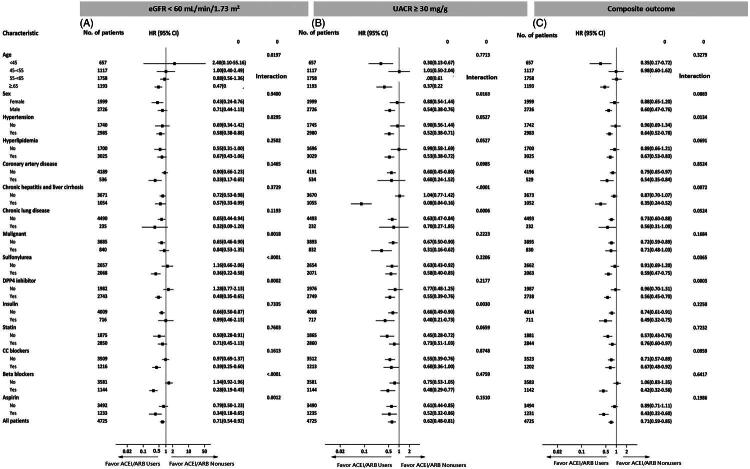
Subgroup analysis of (A) eGFR < 60 mL/min/1.73 m^2^ (B) UACR ≥ 30 mg/g and (C) composite outcome among all patients. In multivariable Cox models, ACEI/ARB use was generally associated with lower risks across most subgroups for all three outcomes, although some stratification factors showed significant interactions.

We performed subgroup analyses stratified by baseline comorbidities and concomitant medications (Figure 2). In multivariable Cox proportional hazards regression models, the differences in risk between ACEI/ARB users and nonusers were consistent across most subgroups. This pattern was observed for all three outcomes, including eGFR <60 mL/min/1.73 m^2^, UACR ≥30 mg/g, and the composite outcome. While some stratification factors demonstrated significant interactions, the overall pattern across outcomes still supported a generally consistent benefit of ACEI/ARB use.

### Sensitivity analysis of blood glucose control

In this sensitivity analysis incorporating HbA1c as a time-dependent covariate (Supplementary Table 1), ACEI/ARB use remained significantly associated with lower risks of eGFR <60 mL/min/1.73 m^2^, UACR ≥30 mg/g, and the composite outcome. These findings indicate that the renal protective effect of RAS blockers persisted even after accounting for dynamic changes in glycemic control.

### Active-comparator analysis: ACEI/ARB versus calcium-channel blockers

Compared with calcium-channel blocker users, ACEI/ARB use was associated with significantly lower risks of eGFR <60 mL/min/1.73 m^2^, UACR ≥30 mg/g, and the composite outcome. These findings support the robustness of the observed renoprotective association against confounding by antihypertensive regimen (Supplementary Table 2).

### Sensitivity analysis stratified by follow-up duration

In the longer follow-up stratum (>1.5 years), ACEI/ARB use was associated with significantly lower risks of eGFR <60 mL/min/1.73 m^2^, UACR ≥30 mg/g, and the composite outcome. In contrast, in the ≤1.5-year stratum these associations did not reach statistical significance, and the point estimates for the eGFR and composite outcomes were close to or above unity. These findings indicate that the renoprotective association of RAS blockade became apparent predominantly with longer follow-up, consistent with the time required for new-onset CKD to develop (Supplementary Table 3).

### Sensitivity analysis using baseline covariates only

In this model, ACEI/ARB use remained significantly associated with lower risks of new-onset UACR ≥30 mg/g and of the composite outcome, whereas its association with eGFR <60 mL/min/1.73 m^2^ was no longer statistically significant. These results indicate that the protective associations for albuminuria and the composite outcome were robust to a baseline-only adjustment, consistent with the principal findings of the study (Supplementary Table 4).

### Sensitivity analysis of potential residual confounder

To evaluate the potential impact of an unmeasured confounder on our findings, we conducted a sensitivity analysis using the R package ‘obsSens’, introducing a hypothetical residual confounder with varying prevalences between the ACEI/ARB and non-ACEI/ARB groups (Supplementary Figure 3A–C) [16]. For instance, when all non-ACEI/ARB subjects were assumed to have this unmeasured confounder (prevalence = 1.0) and no ACEI/ARB users had it (prevalence = 0.0), the association between ACEI/ARB use and reduced risk remained (eGFR <60 mL/min/1.73 m^2^: HR =0.46, the leftmost point on the bottom line in Supplementary Figure 3A). Across a wide range of assumed prevalences of the unmeasured confounder, the associations of ACEI/ARB use with lower risks of eGFR <60 mL/min/1.73 m^2^, UACR ≥30 mg/g, and the composite outcome remained robust.

## Discussion

In this large-scale cohort study including 316,693 individuals with type 2 diabetes, we demonstrated that the use of renin-angiotensin system (RAS) blockers was significantly associated with a reduced risk of developing new-onset CKD in patients with hypertension and normal baseline renal function. This protective effect remained consistent across multiple modeling approaches, including conventional multivariable adjustment, competing risk analysis, and time-dependent Cox models, underscoring the potential role of ACEIs/ARBs in preventing kidney disease progression in this population.

Previous studies evaluating the renal effects of RAS blockade in patients with diabetes and hypertension have yielded heterogeneous findings, likely reflecting differences in study population, comparator treatment, baseline renal status, blood-pressure status, and renal endpoints. In ALLHAT, participants were hypertensive adults aged 55 years or older with at least one additional cardiovascular risk factor [17,18]. Among the 13,101 participants with diabetes, first-step antihypertensive therapy with lisinopril did not show superiority over chlorthalidone for major clinical outcomes, including end-stage kidney disease. However, ALLHAT was primarily designed as a cardiovascular outcomes trial, albuminuria was not systematically measured, and the trial did not specifically enroll patients with preserved kidney function and normoalbuminuria for the purpose of evaluating incident CKD. The neutral renal findings in ALLHAT should therefore be interpreted as addressing a later-stage and clinically different question from that of our study.

UKPDS 39 provides additional context [19]. In this randomized trial, 1,148 hypertensive patients with type 2 diabetes were enrolled, and among those assigned to tight blood-pressure control, captopril and atenolol were compared as active antihypertensive agents. The two treatments achieved similar blood-pressure reduction and showed no clear difference in albuminuria progression, clinical proteinuria, doubling of plasma creatinine, or renal failure, suggesting that, in that setting, blood-pressure lowering itself may have been more important than the specific antihypertensive class. Nevertheless, UKPDS 39 was not designed to evaluate whether RAS blockade prevents the first onset of CKD in patients with preserved baseline kidney function and normoalbuminuria, and its renal endpoints included later manifestations such as overt proteinuria and doubling of plasma creatinine.

Among prior trials, ROADMAP was the closest to our study with respect to the early albuminuria endpoint [9]. ROADMAP enrolled patients with type 2 diabetes and normoalbuminuria and tested whether olmesartan could delay or prevent the first onset of microalbuminuria. Olmesartan significantly delayed the onset of microalbuminuria compared with placebo, whereas doubling of the serum creatinine level occurred with similar frequency in both groups. This pattern is directionally consistent with our findings, in which the association of ACEI/ARB use was more robust for incident albuminuria and the composite CKD endpoint than for the eGFR-based endpoint alone. ROADMAP differed from our study in several respects: it was a randomized placebo-controlled trial of a single ARB, blood pressure was slightly lower in the olmesartan group, and a higher number of fatal cardiovascular events was observed among patients with preexisting coronary heart disease. Nevertheless, it supports the biological and clinical plausibility of early albuminuria prevention with RAS blockade, particularly in patients with higher baseline blood pressure.

Other trials illustrate why findings in early diabetic kidney disease remain difficult to interpret [10]. RASS enrolled normotensive, normoalbuminuric patients with type 1 diabetes and compared enalapril, losartan, and placebo over 5 years; neither active agent significantly improved GFR decline or renal structural outcomes, and the cumulative incidence of microalbuminuria was higher in the losartan group than in the placebo group. Similarly, the trial by Weil et al. studied American Indians with type 2 diabetes and either normoalbuminuria or microalbuminuria at baseline [7]. Losartan did not significantly reduce the GFR-based primary outcome, which occurred in only a small number of participants, and the structural benefit, reflected by a lower mesangial fractional volume, was observed mainly among patients with baseline microalbuminuria rather than normoalbuminuria. Importantly, both RASS and Weil et al. raised a potential signal of harm rather than benefit in normoalbuminuric participants: in Weil et al. losartan was associated with a higher risk of progression to macroalbuminuria among normoalbuminuric patients. A key distinction from our study, however, is the blood-pressure status and indication for treatment. These potential harm signals arose in normotensive or predominantly normotensive cohorts without a compelling antihypertensive indication for RAS blockade, whereas our population comprised patients with type 2 diabetes and new-onset hypertension, in whom ACEIs or ARBs were initiated as antihypertensive therapy. This distinction is clinically relevant because ROADMAP, a trial conducted in a more hypertension-relevant context, showed a stronger albuminuria benefit among patients with higher baseline systolic blood pressure [9].

Taken together, the apparent discrepancies between earlier studies and our findings may be explained, at least in part, by differences in baseline renal status, diabetes type, hypertension status, comparator treatment, and endpoint definition. Trials using later or less frequent renal endpoints, such as doubling of serum creatinine, end-stage kidney disease, or structural change, generally did not demonstrate a clear advantage of RAS blockade in patients with early or preserved kidney function. In contrast, studies focusing on incident albuminuria, particularly ROADMAP, showed a signal more consistent with our albuminuria findings. Our data should therefore not be interpreted as definitive evidence that ACEI/ARB therapy preserves eGFR in all patients with type 2 diabetes and preserved kidney function; rather, they suggest that ACEI/ARB use is associated with a lower risk of early albuminuria development and composite new-onset CKD, whereas the association with the eGFR-based endpoint is less consistent and should be interpreted with caution.

Notably, an interesting divergence emerged across our three outcomes. Whereas the protective associations with new-onset albuminuria and with the composite outcome were consistent across all modeling approaches, the association with the incident eGFR <60 mL/min/1.73 m^2^ endpoint—although significant in the multivariable model and competing-risk analysis—was attenuated and became non-significant in the time-dependent Cox model. This discrepancy merits closer examination. RAS blockade produces a well-recognized acute hemodynamic reduction in eGFR mediated by efferent arteriolar vasodilation; when exposure is modeled as a time-varying variable tracking active treatment, this initial eGFR dip may transiently move some patients across the 60 mL/min/1.73 m^2^ threshold, attenuating the apparent protective effect on this creatinine-based endpoint. This mechanism does not apply to albuminuria, and accordingly the protective associations with new-onset UACR ≥30 mg/g and with the composite outcome persisted in the time-dependent model. Taken together, the renoprotective signal of RAS blockade in this population is most robust for albuminuria and the composite outcome, whereas its effect on the eGFR threshold is more sensitive to how exposure is modeled and warrants cautious interpretation.

Several limitations should be acknowledged. First, the observational, non-randomized design leaves the study open to the biases inherent in such data. Second, the two treatment groups were not identical at baseline; although GBM weighting was used to reconcile these differences, residual confounding from unmeasured factors cannot be excluded. Third, comorbidities were ascertained from administrative claims, raising the possibility of diagnostic misclassification. Fourth, because the study period ended in 2016, GLP-1 receptor agonists and SGLT2 inhibitors were used by very few patients; while this minimizes confounding from these strongly renoprotective agents and allows the independent effect of RAS blockade to be assessed more clearly, it also limits the direct generalizability of our findings to the contemporary treatment era. Validation in cohorts incorporating these newer therapies is warranted. Fifth, the RAS-blockade group was overwhelmingly composed of ARB users, with only a few receiving ACEIs; an adequately powered ACEI-versus-ARB comparison was therefore not feasible, and potential class-specific differences warrant evaluation in future studies.

In this large multicenter retrospective cohort study, ACEI/ARB use was associated with a significantly lower risk of new-onset CKD in patients with type 2 diabetes, new-onset hypertension, preserved baseline kidney function, and no albuminuria. This association was most consistent for incident albuminuria and the composite CKD outcome, whereas the eGFR-based endpoint was less robust in the time-dependent analysis. From a practical clinical perspective, our findings suggest that, when antihypertensive therapy is initiated in patients with type 2 diabetes and preserved kidney function, an ACEI or ARB may be a reasonable preferential choice over other antihypertensive classes, particularly when the prevention of incident albuminuria is a major therapeutic concern. Although blood-pressure targets were not evaluated in our study, lowering blood pressure toward guideline-recommended targets, as tolerated, remains an important component of overall renal and cardiovascular risk reduction. However, because this was an observational study, further prospective studies are needed to confirm these findings and to clarify the optimal timing and appropriate patient selection for RAS blockade in the prevention of new-onset CKD.

## Supplementary Material

Supplementary data.docx

## Data Availability

The data that support the findings of this study are available on request from the corresponding author, [Y.-C. Lee], upon reasonable request. The data are not publicly available due to privacy and ethical restrictions, as they contain information that could compromise the privacy of research participants.
