# Supplementary material for: Prevention of new-onset chronic kidney disease with renin–angiotensin system blockers in type 2 diabetes with preserved kidney function
Source: Ren Fail. 2026 Aug 5;48(1):2711459. doi: 10.1080/0886022X.2026.2711459 (PMC13446052; doi:10.1080/0886022X.2026.2711459)
Supplement: Supplementary file 1 [file IRNF_A_2711459_SM5661.docx]

**Supplementary Figure Legends**

**Supplementary Figure 1. Study flow chart.**

**Supplementary Figure 2. Absolute standardized mean differences of baseline characteristics before and after GBM weighting.** Love plot showing the absolute standardized mean differences (aSMD) for baseline characteristics between the two groups before and after generalized boosted model (GBM) weighting. The vertical dashed line at aSMD = 0.1 indicates the commonly accepted threshold for adequate covariate balance. After GBM weighting, most covariates achieved aSMD values below 0.1, demonstrating substantial improvement in baseline balance compared with the unweighted sample.

 **Supplementary Figure 3. Sensitivity analysis of the hazard ratio (HR) for renal outcomes after adjustment for a hypothetical unmeasured confounder.** This analysis evaluates how the association between ACEI/ARB use and renal outcomes might change under different assumptions about an unmeasured confounding factor. The x-axis represents the prevalence of the hypothetical confounder among ACEI/ARB users, and the colored lines indicate different prevalences of the same confounder among non-users. The y-axis represents the adjusted hazard ratio for each renal outcome (A: eGFR < 60 mL/min/1.73 m², B: UACR ≥ 30 mg/g, C: composite outcome). Across most plausible scenarios, the HR remains below 1.0, indicating that ACEI/ARB users consistently have lower risks than non-users even after accounting for a potential unmeasured confounder. These results suggest that the observed protective association of RAS blockers is robust and unlikely to be fully explained by residual confounding. For example, when all non–ACEI/ARB users were assumed to have the unmeasured confounder (prevalence = 1.0) and ACEI/ARB users did not have it (prevalence = 0.0), the estimated HR for the composite renal outcome remained protective (HR = 0.45; corresponding to the leftmost point on the bottom line in panel C).

Supplementary Table 1. Evaluating the influence of dynamic HbA1c levels on RAS blocker‑associated renal outcomes using a time‑dependent Cox model

|  | All | | |  | eGFR≥90 mL/min/1.73 m^2^ | | |  | 60 mL/min/1.73 m^2^  eGFR  <90 mL/min/1.73 m2 | | |
| --- | --- | --- | --- | --- | --- | --- | --- | --- | --- | --- | --- |
|  | HR | 95% CI | P Value |  | HR | 95% CI | P Value |  | HR | 95% CI | P Value |
| eGFR<60 mL/min/1.73 m^2^ | 0.69 | (0.53 – 0.89) | 0.0048 |  | 0.56 | (0.34 – 0.91) | 0.0205 |  | 0.66 | (0.48 – 0.91) | 0.0111 |
| UACR ≥ 30 | 0.62 | (0.47 – 0.80) | 0.0004 |  | 0.66 | (0.48 – 0.91) | 0.0110 |  | 0.41 | (0.26 – 0.65) | 0.0001 |
| Composite outcome | 0.71 | (0.60 – 0.85) | 0.0002 |  | 0.75 | (0.57 – 0.99) | 0.0389 |  | 0.63 | (0.50 – 0.81) | 0.0003 |

Time-dependent Cox proportional hazards models were adjusted for age, sex, baseline eGFR, baseline UACR, DM duration, comorbidity (including variables with aSMD0.1), medication (including variables with aSMD0.1) and laboratory data. The primary exposure of interest was ACEI/ARB use; HbA1c was included as a time-varying covariate to account for dynamic glycemic control during follow-up.

Supplementary Table 2. Active-comparator analysis: adjusted hazard ratios (HRs) for renal outcomes in ACEI/ARB users versus CC blockers users.

| Type of Treatment  (No. of Patients) | Study Outcome, HR (95% CI) |
| --- | --- |
| eGFR < 60 mL/min/1.73 m^2^ |  |
| CC blockers |  |
| ACEI/ARB user | 0.42 (0.33 – 0.52) |
| UACR ≥ 30 |  |
| CC blockers |  |
| ACEI/ARB user | 0.76 (0.59 – 0.97) |
| Composite outcome |  |
| CC blockers |  |
| ACEI/ARB user | 0.53 (0.45 – 0.63) |

Adjusted for age, sex, baseline eGFR, baseline UACR, DM duration, comorbidity (including variables with aSMD0.1), medication (including variables with aSMD0.1) and laboratory data

ACEI denotes angiotensin-converting enzyme inhibitors, ARB angiotensin-receptor blocker, and CC blockers calcium channel blockers

Supplementary Table 3. GBM-weighted adjusted hazard ratios (HRs) for renal outcomes in ACEI/ARB users versus nonusers, stratified by follow-up duration (≤1.5 vs >1.5 years)

| Type of Treatment | Study Outcome, HR (95% CI) | |
| --- | --- | --- |
|  | 1.5 years | >1.5 years |
| eGFR < 60 mL/min/1.73 m^2^ |  |  |
| Nonuser |  |  |
| ACEI/ARB user | 1.89 (0.91 – 3.91) | 0.65 (0.49 – 0.86) |
| UACR ≥ 30 |  |  |
| Nonuser |  |  |
| ACEI/ARB user | 0.84 (0.33 – 2.15) | 0.63 (0.48 – 0.83) |
| Composite outcome |  |  |
| Nonuser |  |  |
| ACEI/ARB user | 1.23 (0.71 – 2.15) | 0.68 (0.56 – 0.82) |

Adjusted for age, sex, baseline eGFR, baseline UACR, DM duration, comorbidity (including variables with aSMD0.1), medication (including variables with aSMD0.1) and laboratory data

ACEI denotes angiotensin-converting enzyme inhibitors, and ARB angiotensin-receptor blocker

Supplementary Table 4. Sensitivity analysis using baseline covariates only: GBM-weighted adjusted hazard ratios (HRs) for renal outcomes in ACEI/ARB users versus nonusers.

| Type of Treatment  (No. of Patients) | Study Outcome, HR (95% CI) |
| --- | --- |
| eGFR < 60 mL/min/1.73 m^2^ |  |
| Nonuser |  |
| ACEI/ARB user | 0.95 (0.83 – 1.09) |
| UACR ≥ 30 |  |
| Nonuser |  |
| ACEI/ARB user | 0.63 (0.55 – 0.72) |
| Composite outcome |  |
| Nonuser |  |
| ACEI/ARB user | 0.81 (0.69 – 0.96) |

Adjusted for age, sex, baseline eGFR, baseline UACR, DM duration, comorbidity (including variables with aSMD0.1), medication (including variables with aSMD0.1) and laboratory data

ACEI denotes angiotensin-converting enzyme inhibitors, and ARB angiotensin-receptor blocker
